# Cell Tropism-Related Gene Signatures in MDD Identified by Transcriptomic Analysis and Rat Model Validation

**DOI:** 10.3390/ijms27198780

**Published:** 2026-09-30

**Authors:** Jie Min, Rongrong Jiang, Qiuyue Xu, Yu Chen, Qianyu Han, Chuanlin Yang, Lin Lin, Guihua Xu

**Affiliations:** School of Nursing, Nanjing University of Chinese Medicine, Nanjing 210023, China; 202411122@njucm.edu.cn (J.M.); jrr880107@njucm.edu.cn (R.J.); qiuyuexu@njucm.edu.cn (Q.X.); 202611248@njucm.edu.cn (Y.C.); 065024307@njucm.edu.cn (Q.H.); 065024345@njucm.edu.cn (C.Y.)

**Keywords:** major depressive disorder, chemotaxis, biomarkers, machine learning, neuroinflammation

## Abstract

Major depressive disorder (MDD) is associated with immune dysregulation, but the contribution of chemotaxis-related genes remains incompletely understood. This study aimed to identify chemotaxis-related signatures in MDD and evaluate their diagnostic relevance. Two peripheral-blood microarray datasets (GSE76826 and GSE38206) were integrated after batch correction, and differentially expressed genes were intersected with the GOBP_TAXIS gene set. Hub genes were selected using random forest and SVM-RFE and evaluated by logistic regression, ROC, calibration, and decision curve analyses. Immune-cell abundance, molecular subtypes, single-nucleus expression, and in silico gene-knockout effects were further examined, followed by qRT-PCR and Western blot validation in a CUMS rat model. The integrated cohort included 21 controls and 29 MDD cases. GOBP_TAXIS activity was increased in MDD (*p* < 0.001), and seven candidate genes yielded three upregulated hub genes (*CXCL2*, *NRG1*, and *C3AR1*). The fixed three-gene model yielded an exploratory AUC of 0.889 in the development cohort. All three genes correlated positively with monocytes/neutrophils and negatively with T cells. Single-cell analysis revealed cell-specific enrichment, while in silico knockout simulations predicted transcriptional changes involving RORB and NTNG1 following virtual perturbation of *CXCL2* and *NRG1*. Animal experiments confirmed consistent upregulation at both transcriptional and translational levels. *CXCL2*, *NRG1*, and *C3AR1* represent candidate chemotaxis-related biomarkers for MDD and are associated with distinct immune-cell abundance patterns and inflammatory signatures, warranting further functional validation.

## 1. Introduction

Major depressive disorder (MDD) is a common and highly disabling psychiatric illness characterized by persistent low mood, anhedonia, sleep disturbances, cognitive impairment, and diminished social functioning [1,2]. The Global Burden of Disease Study indicates that MDD affects approximately 332 million individuals, accounting for 4% of the global population, and represents one of the leading causes of years lived with disability (YLD) [3,4]. The World Health Organization projects that by 2030, depressive disorders will rank first in global disease burden [5,6]. Despite the escalating societal impact of MDD, its pathogenesis remains incompletely understood. Current perspectives suggest that MDD may arise from the combined effects of monoamine neurotransmitter imbalance, hypothalamic-pituitary-adrenal axis dysfunction, neuroinflammation, and impaired neuroplasticity [7,8,9]. First-line clinical treatment relies primarily on monoamine-based antidepressants; however, approximately one-third of patients show inadequate response to conventional pharmacotherapy and progress to treatment-resistant depression [10,11]. This observation implies that monoaminergic system abnormalities do not encompass the entirety of MDD pathogenesis, and existing therapeutic strategies have yet to fully address other critical pathological processes such as neuroinflammation and neuroplasticity deficits.

The chemotaxis pathway serves as a crucial link connecting peripheral immune activation with central neuroinflammation. Specifically, the chemokine-receptor signaling axis mediates the directed migration of monocytes, neutrophils, and T lymphocytes across the blood–brain barrier, triggering aberrant immune cell infiltration within brain tissue and subsequently amplifying neurotoxic inflammatory cascades [12,13]. In recent years, the pivotal role of such immune-neural interactions in MDD pathogenesis has garnered increasing attention. Clinical studies have demonstrated significantly elevated levels of pro-inflammatory cytokines including *IL-6*, *TNF-α*, and *CRP* in the peripheral blood of MDD patients, alongside abnormal proportions of neutrophils and monocyte lineage cells [14,15,16]. These peripheral immune system abnormalities represent the potential driving force that activates the aforementioned chemotaxis pathways and induces central inflammation.

Neuroimaging and postmortem studies have further revealed pronounced neuroinflammatory pathological alterations in the prefrontal cortex and anterior cingulate cortex of MDD patients, including microglial and astrocytic activation as well as perivascular macrophage infiltration [17,18,19]. Notably, this central inflammation does not occur in isolation. Research has confirmed that peripheral immune cells can migrate toward the central nervous system via a compromised blood–brain barrier, directly interacting with glial cells and neurons to drive neuroinflammatory cascades and synaptic dysfunction [20,21,22]. Within this process, cell chemotaxis, as the core biological mechanism governing directed immune cell migration, plays an essential bridging role [21,22]. Previous transcriptomic studies have identified upregulated expression of immune-related pathways including chemokine receptor activity, chemotaxis, and cytokine biosynthesis in MDD [23,24,25], suggesting that dysregulated chemotaxis may constitute an important component of the immune pathological mechanisms underlying MDD.

Single-cell RNA sequencing (scRNA-seq) technology enables the resolution of cellular heterogeneity and transcriptional states at the single-cell level, thereby revealing the molecular characteristics of distinct cell populations in MDD. Microarray-based transcriptomic profiling enables the assessment of gene expression patterns across bulk clinical samples. Integrating microarray expression profiling with single-cell RNA sequencing provides complementary information for characterizing aberrant expression patterns of chemotaxis-related genes in MDD and exploring their cell-type-specific distributions. Therefore, this study aimed to screen GOBP_TAXIS-related differentially expressed genes in MDD using GEO microarray datasets, construct a diagnostic prediction model based on machine-learning algorithms, and further characterize the cell-specific expression patterns and computational perturbation-associated transcriptional changes of hub genes using single-cell RNA sequencing data. Furthermore, Sprague–Dawley rats were employed for in vivo experimental validation in the present study due to their well-defined genetic background and widespread use as a model in depression research. The CUMS paradigm can recapitulate core clinical features of human MDD, including anhedonia, psychomotor retardation, and behavioral despair, as well as neuroinflammatory alterations in the prefrontal cortex, showing parallelism with human immune dysregulation and thus offering a translational platform for validating chemotaxis-related genes identified by transcriptomic screening.

## 2. Results

### 2.1. Upregulation of GOBP_TAXIS-Related Genes in MDD

**Figure 1:**
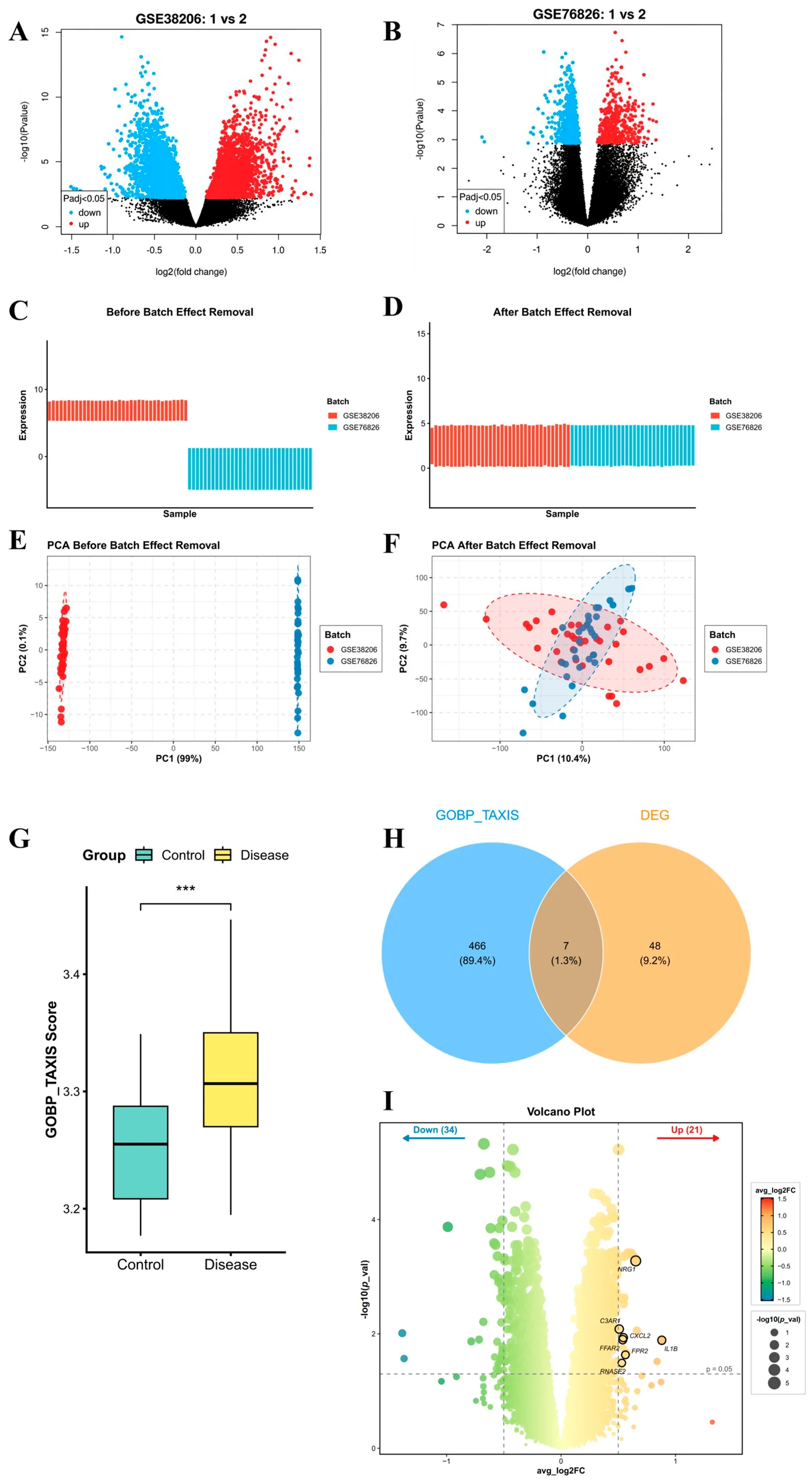
**Identification of GOBP_TAXIS-related differentially expressed genes in MDD.** (**A**) Volcano plot of differentially expressed genes in the GSE38206 dataset; (**B**) Volcano plot of differentially expressed genes in the GSE76826 dataset; (**C**) Box plot of expression value distribution for two GEO datasets before batch effect correction; (**D**) Box plot of expression value distribution for two GEO datasets after batch effect correction; (**E**) Principal component analysis of two datasets before batch effect correction; (**F**) Principal component analysis of two datasets after batch effect correction; (**G**) ssGSEA enrichment scores of GOBP_TAXIS-related gene sets between the control group and MDD group; (**H**) Venn diagram of the intersection between differentially expressed genes and GOBP_TAXIS-related genes; (**I**) Volcano plot of differentially expressed genes between MDD and normal tissues. Differential-expression statistics were obtained using the limma empirical Bayes-moderated linear model, with *p* values adjusted using the false discovery rate (FDR) method. Genes with |log2FC| > 0.5 and FDR-adjusted *p* < 0.05 were considered differentially expressed. Group differences in the GOBP_TAXIS enrichment score in panel G were evaluated using a two-sided Wilcoxon rank-sum test (non-parametric). *** *p* < 0.001.

A substantial number of differentially expressed genes were identified in both GEO microarray datasets (GSE38206 and GSE76826), with roughly balanced numbers of upregulated and downregulated genes. In GSE38206, some differentially expressed genes exhibited higher statistical significance (−log10(*p* value) > 10) (Figure 1A). In GSE76826, the fold change range was broader, with log2FC approximately −2 to 2 (Figure 1B). Both datasets collectively indicated significant transcriptomic expression disturbances in MDD patients. After correction using the sva package, PCA demonstrated effective elimination of batch effects, with the variance contribution rate of PC1 decreasing from 99% to 10.4% (Figure 1C–F). Fifty-five differentially expressed genes (DEGs) were identified, and intersection with 473 GOBP_TAXIS-related genes yielded seven candidate genes (Figure 1H). Among these, *NRG1*, *IL1B*, *CXCL2*, *FFAR2*, *C3AR1*, *FPR2*, and *RNASE2* were all upregulated (Figure 1I). Furthermore, ssGSEA results showed that GOBP_TAXIS enrichment scores were significantly higher in the MDD group than in the control group (*p* < 0.001) (Figure 1G).

**Figure 2:**
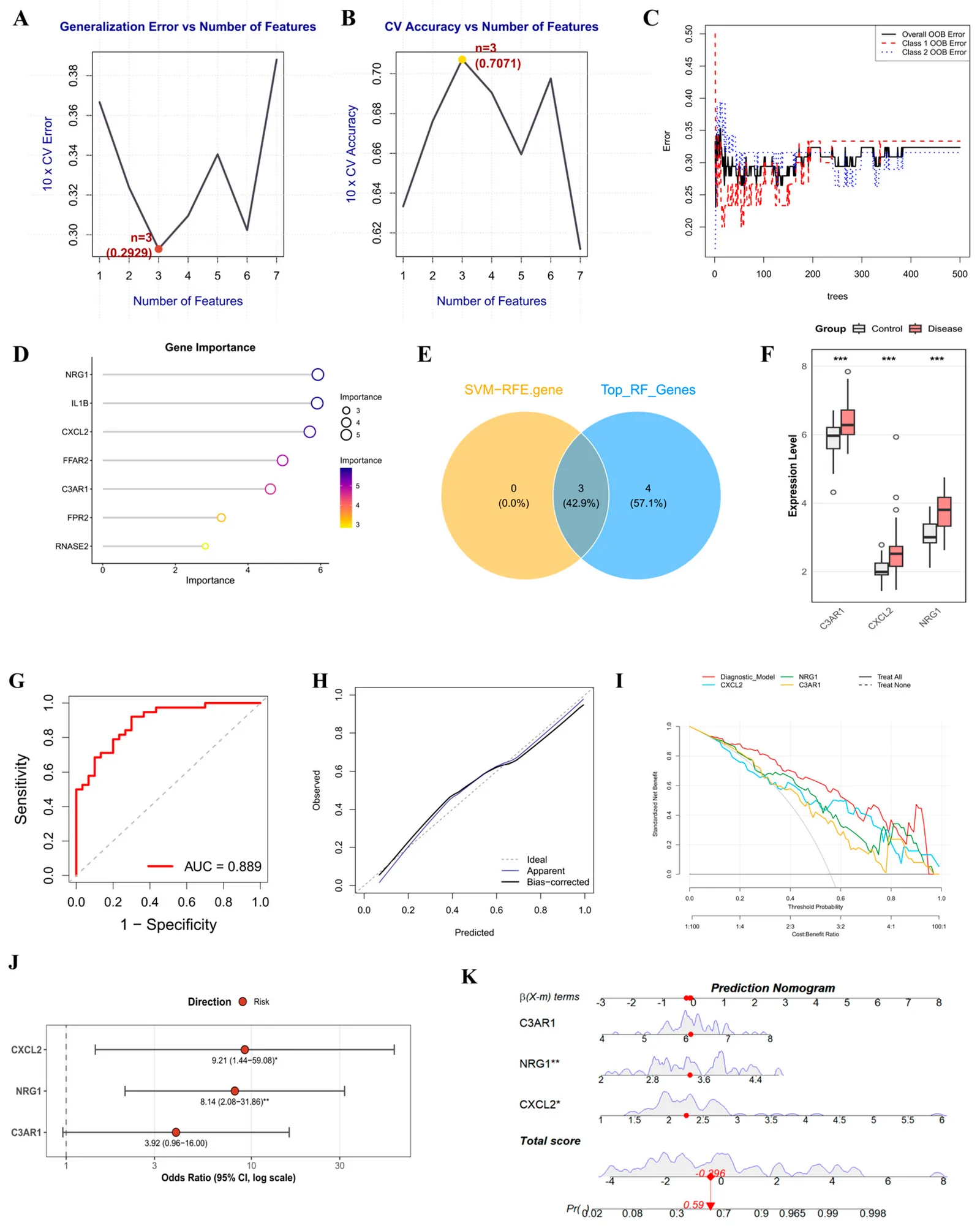
**Screening of GOBP_TAXIS-related hub genes based on machine learning algorithms and construction and evaluation of a hub gene-based MDD diagnostic model.** (**A**) SVM-RFE generalization error curve; (**B**) SVM-RFE cross-validation accuracy curve; (**C**) Random forest OOB error curve; (**D**) Random forest feature importance ranking; (**E**) Venn diagram of intersecting genes from two algorithms; (**F**) Comparison of hub gene expression levels between the control group and MDD group; (**G**) ROC curve of the multivariable logistic regression model; (**H**) Model calibration curve; (**I**) Decision curve analysis; (**J**) Forest plot of multivariable analysis; (**K**) Nomogram for MDD risk prediction. Gene-expression differences in panel (**F**) were assessed using two-sided Welch’s *t*-tests (parametric). The calibration curve was internally assessed using 800 bootstrap resamples, and decision curve analysis used 100 bootstrap resamples. Odds ratios and 95% confidence intervals in panel J were derived from multivariable logistic regression, with *p* values based on Wald tests. * *p* < 0.05, ** *p* < 0.01, and *** *p* < 0.001.

Subsequently, feature selection was performed using a combination of SVM-RFE and random forest algorithms. When the feature number was set to 3, the SVM-RFE model achieved the highest cross-validation accuracy (0.7071) and the lowest error (0.2929) (Figure 2A,B). Random forest importance ranking was *NRG1*, *IL1B*, *CXCL2*, *FFAR2*, *C3AR1*, *FPR2*, and *RNASE2* (Figure 2C,D). The two algorithms jointly identified three overlapping robust feature genes (*CXCL2*, *NRG1*, *C3AR1*) (Figure 2E), and *CXCL2*, *NRG1*, and C3AR1 were significantly upregulated in the MDD group (Figure 2F). These three GOBP_TAXIS-related genes were ultimately designated as the core features for constructing the MDD diagnostic model.

### 2.2. Identification of GTAGs-DEGs

A multivariable logistic regression model was constructed using the three selected hub genes (*CXCL2*, *NRG1*, and *C3AR1*). In the development cohort, ROC curve analysis yielded an AUC of 0.889 (Figure 2G), indicating preliminary discriminatory ability. Bootstrap resampling was used to internally assess the fixed three-gene model after feature selection. Because the upstream feature-selection procedures were not repeated within each bootstrap resample, this AUC should be regarded as an exploratory internally assessed estimate and may be subject to optimism. In the calibration curve, predicted probabilities were generally aligned with observed probabilities within the development cohort (Figure 2H). Decision curve analysis showed that, across a range of threshold probabilities, the standardized net benefit of the three-gene model exceeded that of the single-gene models and the “Treat All” and “Treat None” strategies within the present dataset (Figure 2I). These findings support further evaluation of the model in independent cohorts rather than establishing its clinical utility. Multivariable analysis revealed that *CXCL2* (OR = 9.21, 95%CI 1.44–59.08, *p* < 0.05) and *NRG1* (OR = 8.14, 95%CI 2.08–31.86, *p* < 0.01) were independent risk factors for MDD onset (Figure 2K). The predictive nomogram constructed accordingly incorporated all three genes, calculating a total score based on each gene’s expression level and converting it to individual MDD probability. The final probability range fluctuated between 0.02 and 0.998. When a patient’s total score for the three hub genes was −0.296, the predicted probability of developing MDD was 0.59 (59%) (Figure 2K).

### 2.3. Immune-Cell Abundance Association Analysis

#### Correlation Analysis Between the Three Hub Genes and 22 Immune Cell Infiltration Types

**Figure 3:**
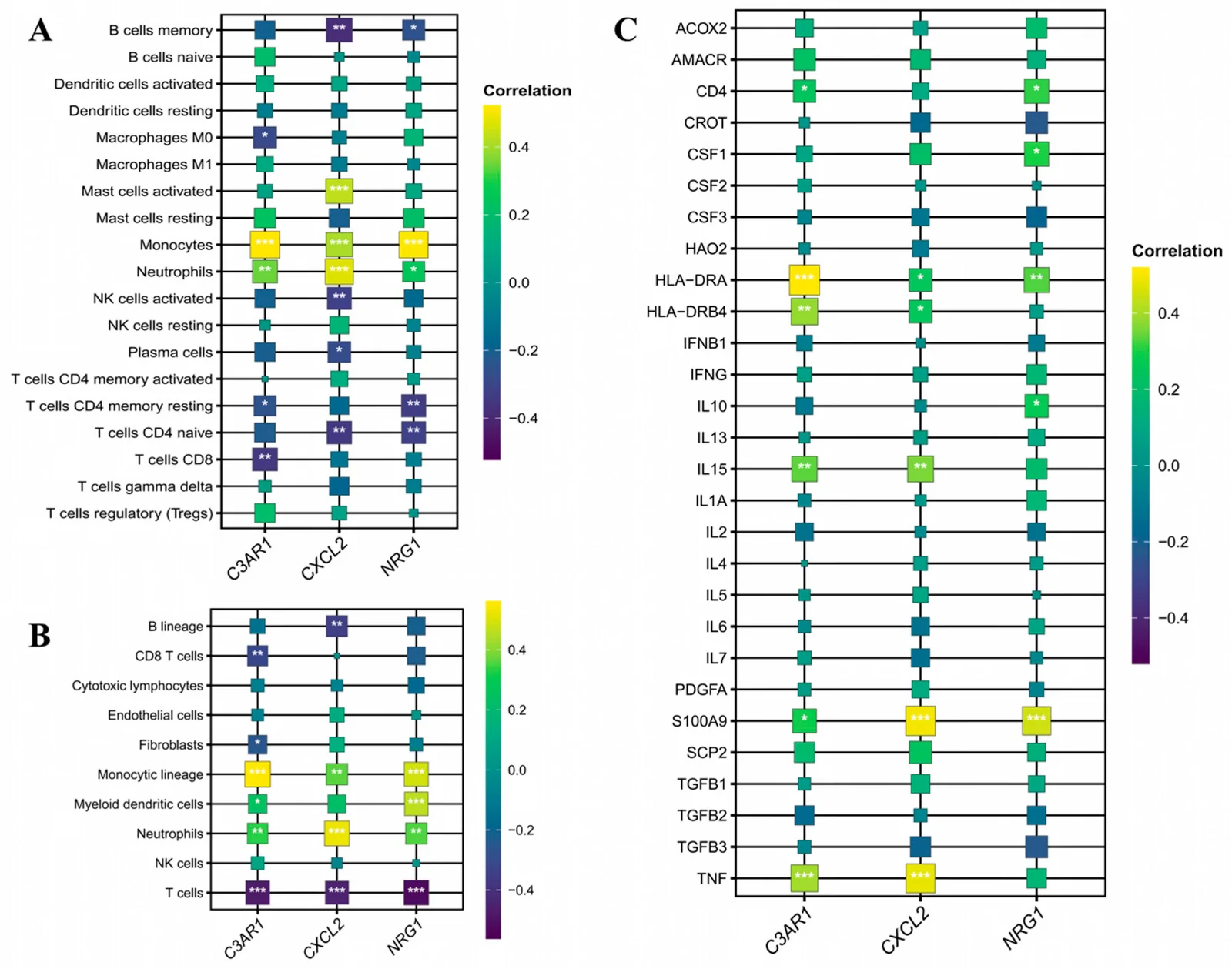
**Associations between hub gene expression, computationally estimated immune-cell abundance, and inflammatory mediators.** (**A**) Correlation heatmap of hub genes with the relative proportions of 22 immune-cell types estimated using CIBERSORT; (**B**) correlation heatmap of hub genes with immune-cell abundance scores estimated using MCP-counter; (**C**) correlation heatmap of hub genes with the expression of 28 inflammation-related factors. Spearman’s rank correlation test (non-parametric) was used for all correlation analyses in panels (**A**–**C**). * *p* < 0.05, ** *p* < 0.01, and *** *p* < 0.001.

Correlation analysis between the three hub genes and computationally estimated immune-cell abundance was performed using CIBERSORT and MCP-counter. CIBERSORT analysis showed that *C3AR1* expression was positively correlated with the estimated proportions of monocytes and neutrophils and negatively correlated with CD8 T cells and other lymphocyte subsets. *CXCL2* expression was positively correlated with activated mast cells, monocytes, and neutrophils and negatively correlated with memory B cells, naïve CD4 T cells, and other lymphocyte subsets. *NRG1* expression was positively correlated with monocytes and negatively correlated with resting memory CD4 T cells, naïve CD4 T cells, and other T-cell subsets(Figure 3A). Consistently, MCP-counter analysis showed that *C3AR1*, *CXCL2*, and *NRG1* were positively correlated with estimated monocytic-lineage and neutrophil abundance (*p* < 0.01) and negatively correlated with estimated T-cell abundance (*p* < 0.001)(Figure 3B). Further analysis of 28 inflammation-related factors (Figure 3C) revealed extensive associations between hub genes and various immune regulatory molecules. For instance, *C3AR1* was significantly positively correlated with *HLA-DRA*, *HLA-DRB4*, *IL15*, and *TNF* (*p* < 0.01). *CXCL2* was significantly positively correlated with *IL15*, *S100A9*, and *TNF* (*p* < 0.01). *NRG1* was significantly positively correlated with *HLA-DRA* and *S100A9* (*p* < 0.01). Collectively, these findings demonstrate statistical associations between hub-gene expression, computationally estimated immune-cell abundance, and inflammatory-mediator expression. They do not establish direct effects of these genes on immune-cell migration, infiltration, or regulation.

### 2.4. Unsupervised Clustering Comparison of Clinical and Immunological Characteristics

**Figure 4:**
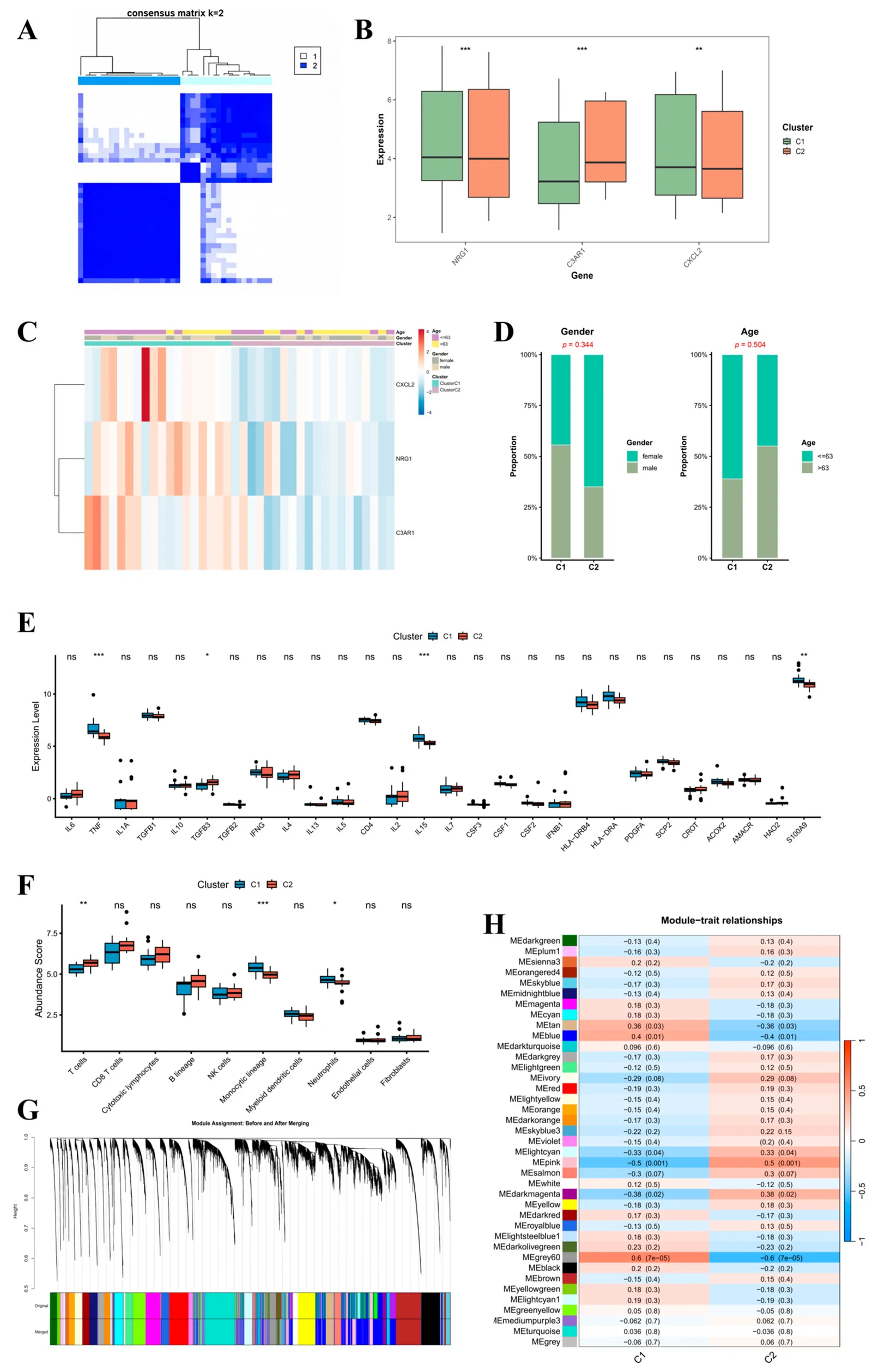
**Consensus clustering of MDD based on hub genes, comparison of clinical and immune characteristics between subtypes, and weighted gene co-expression network analysis (WGCNA).** (**A**) Consensus clustering matrix heatmap at k = 2. (**B**) Comparison of hub gene expression between the two molecular subtypes; P values were calculated using Welch’s two-sample *t*-test. (**C**) Heatmap of hub gene expression and clinical-feature distribution; associations between molecular subtype and categorical clinical characteristics were evaluated using Pearson’s chi-square test. (**D**) Comparison of gender and age-category composition between the two molecular subtypes; P values were calculated using Pearson’s chi-square test. (**E**) Comparison of inflammation-related factor expression between the two molecular subtypes; P values were calculated using the two-sided Wilcoxon rank-sum test. (**F**) Comparison of MCP-counter-estimated immune-cell abundance between the two molecular subtypes; P values were calculated using the two-sided Wilcoxon rank-sum test. (**G**) Gene hierarchical clustering and dynamic tree cutting in WGCNA. (**H**) Module-trait relationship heatmap; correlations between module eigengenes and molecular subtypes were assessed using Pearson correlation, with P values calculated using the WGCNA corPvalueStudent function. Statistical significance is indicated as * *p* < 0.05, ** *p* < 0.01, and *** *p* < 0.001.

Consensus clustering analysis based on the three hub genes (*NRG1*, *C3AR1*, *CXCL2*) stratified MDD samples into two subtypes (Cluster 1 and Cluster 2). The consensus matrix heatmap demonstrated favorable clustering stability at k = 2 (Figure 4A). Differential expression analysis of hub genes between the two subtypes revealed that *C3AR1* was significantly upregulated in Cluster 2 (*p* < 0.001), whereas *NRG1* and *CXCL2* were significantly downregulated in Cluster 2 (*p* < 0.01) (Figure 4B). Clinical characteristic analysis showed no statistically significant differences in gender (*p* = 0.344) or age (*p* = 0.504) distribution between the two clusters (Figure 4C,D), indicating that this classification primarily reflected molecular characteristic differences rather than demographic bias. Inflammatory factor expression profiling demonstrated significant differences in *TNF*, *TGFB3*, *IL15*, and *S100A9* between C1 and C2 (*p* < 0.05) (Figure 4E). Further comparison of immune microenvironment characteristics between the two subtypes using MCP-counter analysis revealed that T cell abundance was significantly higher in Cluster 2 than in Cluster 1 (*p* < 0.01), while Monocytic lineage and Neutrophil abundance were significantly higher in Cluster 1 than in Cluster 2 (*p* < 0.05) (Figure 4F). These findings indicate that MDD molecular subtypes defined by hub-gene expression were associated with distinct computationally estimated immune-cell abundance patterns and inflammatory-factor expression profiles, including differences in T cells, monocytic-lineage cells, and neutrophils. WGCNA analysis identified multiple gene co-expression modules (Figure 4G). Module-trait association analysis showed that MEgrey60 (r = 0.6, *p* = 7 × 10^−5^) was significantly positively correlated with the Cluster 1 subtype (Figure 4H), suggesting that this module may carry key biological information related to MDD subtypes.

### 2.5. Establishment of Co-Expression Network and GO/KEGG Enrichment Analysis

**Figure 5:**
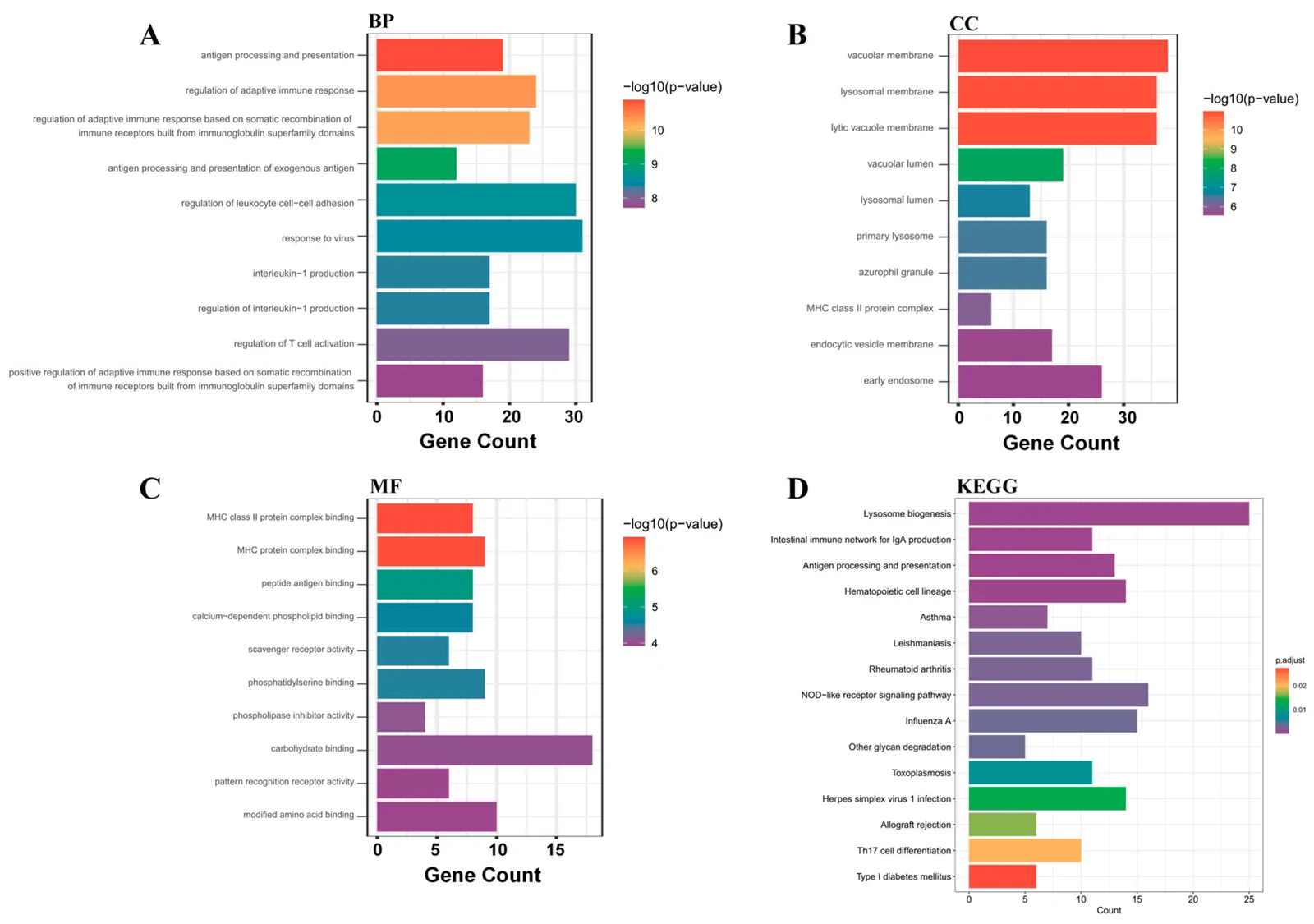
**Functional enrichment analysis of key module genes.** (**A**) Bubble plot of GO biological process (BP) enrichment analysis; (**B**) Bubble plot of GO cellular component (CC) enrichment analysis; (**C**) Bubble plot of GO molecular function (MF) enrichment analysis; (**D**) Bubble plot of KEGG pathway enrichment analysis. GO and KEGG over-representation analyses were performed using the clusterProfiler package with hypergeometric testing and Benjamini–Hochberg correction for multiple comparisons. Bubble size indicates gene count, and color represents enrichment significance. These analyses are not conventional parametric or non-parametric between-group comparisons.

Genes within the MEgrey60 module were imported into the “ClusterProfiler” package for functional enrichment analysis. GO analysis showed that biological processes (BP) were mainly enriched in immune-related terms, including antigen processing and presentation, regulation of adaptive immune response, response to virus, regulation of leukocyte cell–cell adhesion, and regulation of T cell activation, with response to virus and regulation of leukocyte cell–cell adhesion involving the largest number of genes. Cellular components (CC) were primarily localized to lysosome-related structures such as vacuolar membrane, lysosomal membrane, and lytic vacuolar membrane, with the highest enrichment degree (Figure 5B). Molecular functions (MF) were dominated by MHC protein complex binding, peptide antigen binding, and carbohydrate binding (Figure 5C). KEGG pathway analysis (Figure 5D) revealed that these genes were significantly enriched in multiple immune-related pathways, including lysosome biogenesis, antigen processing and presentation, NOD-like receptor signaling pathway, influenza A, and Th17 cell differentiation, with lysosome biogenesis containing the largest number of enriched genes. These results indicate that key module genes primarily participate in antigen processing and presentation, adaptive immune response, and lysosome-related functions, suggesting their potentially important roles in the immunoinflammatory mechanisms of MDD.

### 2.6. Integration, Clustering, and Distribution of Hub Genes in Single-Cell Data

**Figure 6:**
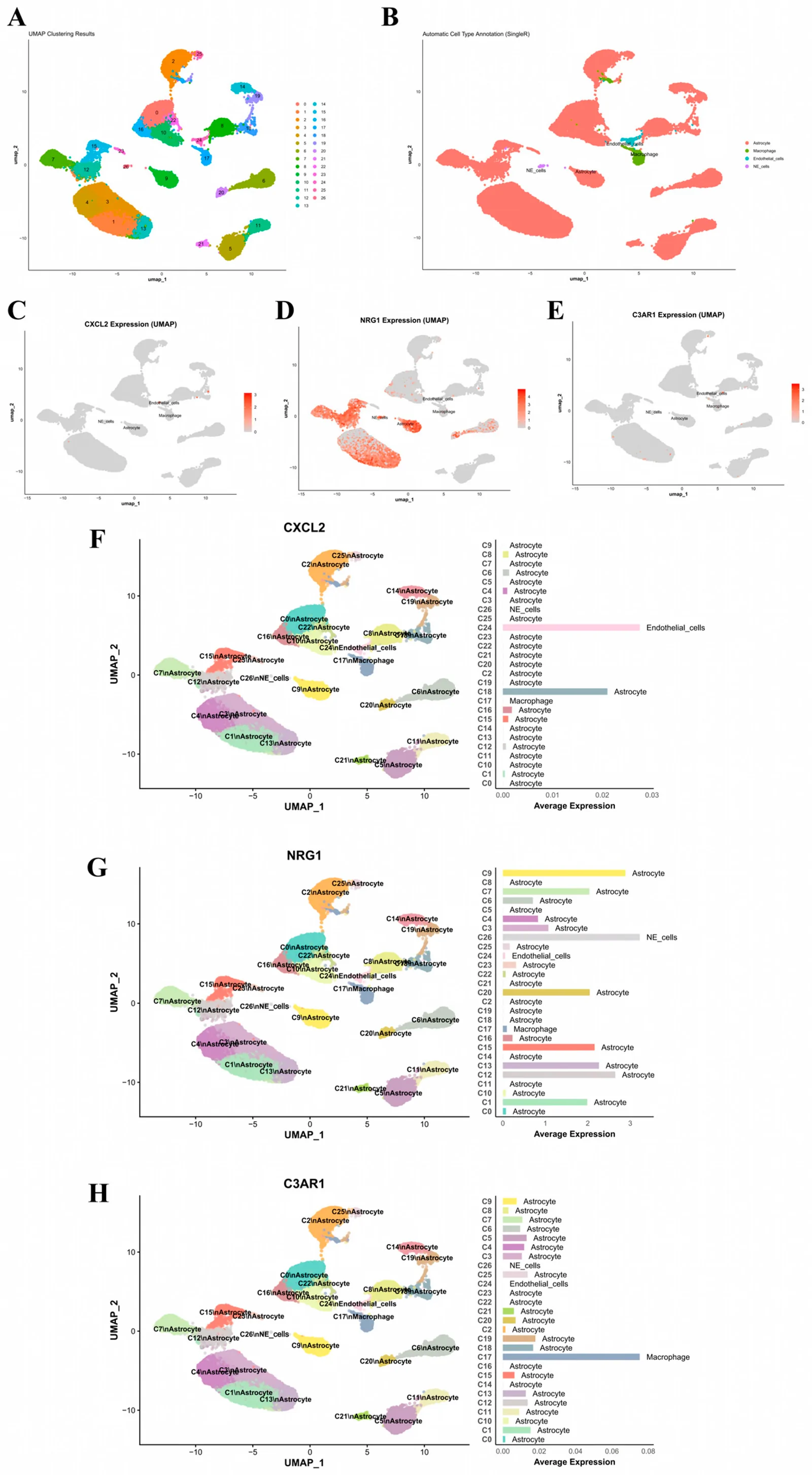
**Cell type annotation and hub gene expression distribution in single-cell data.** (**A**) UMAP clustering result plot; (**B**) Automated cell type annotation plot; (**C**) *CXCL2* expression feature plot; (**D**) *NRG1* expression feature plot; (**E**) *C3AR1* expression feature plot; (**F**) Expression distribution of *CXCL2* across cell subclusters; (**G**) Expression distribution of *NRG1* across cell subclusters; (**H**) Expression distribution of *C3AR1* across cell subclusters.

After quality control and Harmony batch correction, UMAP dimensionality reduction analysis of single-cell sequencing data identified 26 cell clusters (Figure 6A). SingleR automatic annotation classified these into four major cell types: Astrocyte, neuroendocrine cells (NE_cells), endothelial cells (Endothelial_cells), and macrophages (Macrophage) (Figure 6B), with the Astrocyte subpopulation being the absolutely dominant group. Cell-specific expression analysis of hub genes showed that *CXCL2* was primarily enriched in Endothelial_cells (C24) and certain Astrocyte subclusters (C18) (Figure 6C,F). *NRG1* was widely and highly expressed in Astrocyte subclusters, with particularly significant expression in C9, C12, and other subclusters (Figure 6D,G). *C3AR1* expression was relatively dispersed, with low-level expression detectable in Macrophage (C17) and partial Astrocyte subclusters (Figure 6E,H). These results suggest that *NRG1* may serve as an Astrocyte-specific marker, *CXCL2* is mainly derived from Endothelial_cells, and *C3AR1* may participate in immune regulation mediated by Macrophage and Astrocyte. The three hub genes collaboratively participate in GOBP_TAXIS-related pathological processes in MDD through distinct cell types.

**Figure 7:**
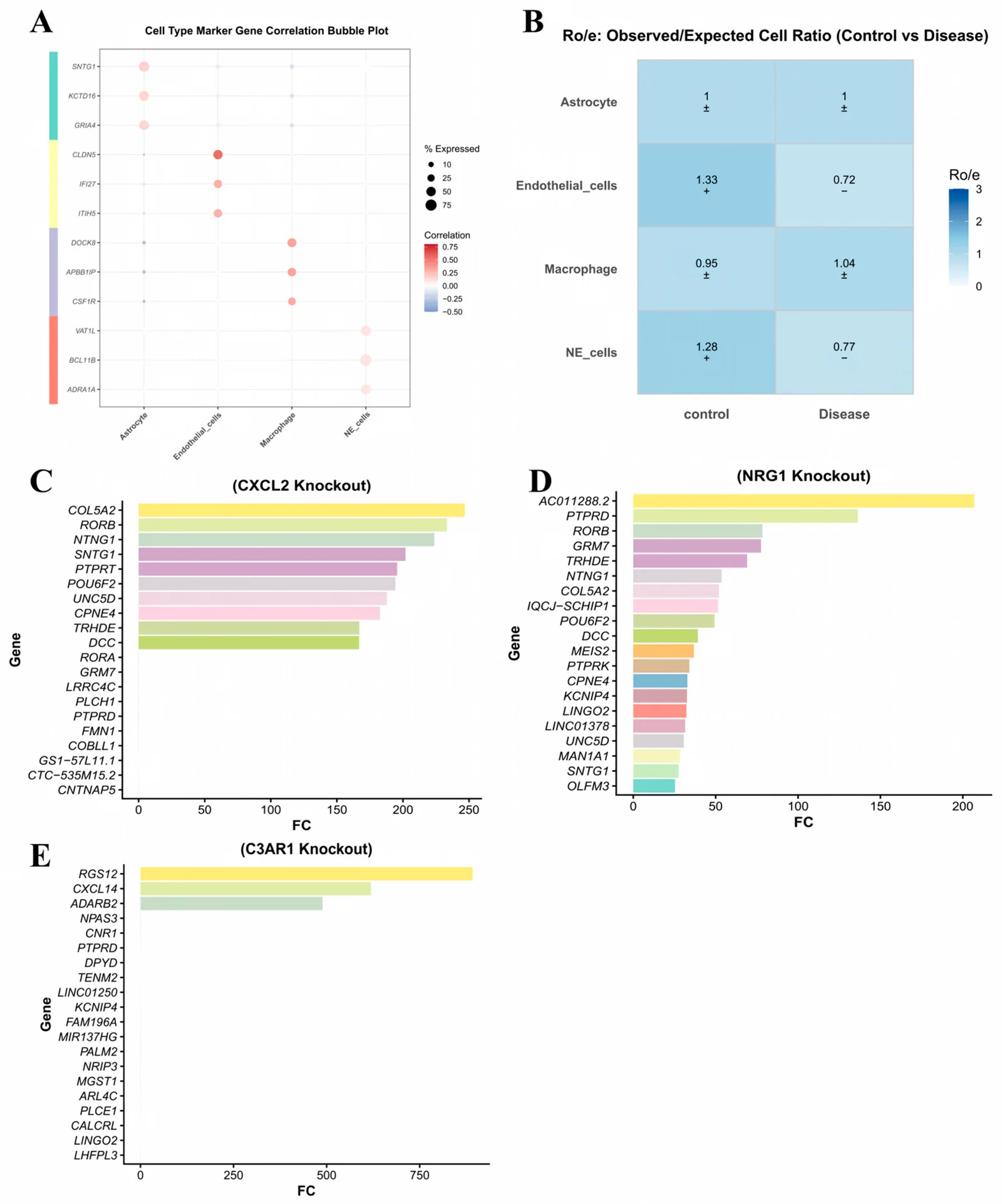
**Validation of cell type annotation at the single-cell level, cell-specific expression of hub genes, and in silico gene knockout simulation analysis.** (**A**) Bubble plot of cell type marker gene correlation; (**B**) Ro/e heatmap of disease-control cell proportions. In the Ro/e matrix, “+” indicates an observed/expected cell ratio greater than 1, denoting relative enrichment of the corresponding cell type; “-” indicates a ratio less than 1, denoting relative depletion; and “±” indicates a ratio close to 1, denoting no substantial deviation from the expected cell proportion. Ro/e, ratio of observed to expected cell numbers; (**C**) Predicted transcriptional changes following in silico *CXCL2* knockout; (**D**) Predicted transcriptional changes following in silico *NRG1* knockout; (**E**) Predicted transcriptional changes following in silico *C3AR1* knockout. Genes with adjusted *p* < 0.05 were considered statistically significant in the virtual-knockout analysis. These analyses were computational perturbation analyses.

Cell type marker gene analysis revealed *SNTG1*, *KCTD16*, and *GRIA4* as characteristic Astrocyte markers, *CLDN5*, *IFI27*, and *ITIH5* as Endothelial_cells markers, *DOCK8*, *APBB1IP*, and *CSF1R* as Macrophage markers, and *VAT1L*, *BCL11B*, and *ADORA1A* as NE_cells markers (Figure 7A). Disease-control comparison showed that Endothelial_cells (Ro/e = 1.33) and NE_cells (Ro/e = 1.28) were significantly enriched in the control group, with reduced proportions in the MDD group. Astrocyte and Macrophage proportions were similar between the two groups (Figure 7B), suggesting a possible relative reduction of Endothelial_cells and NE_cells in MDD patients, while Astrocyte proportions remained relatively stable.

In silico gene knockout simulation using scTenifoldKnk identified predicted transcriptional responses following virtual perturbation of each hub gene. Following simulated *CXCL2* knockout, *COL5A2*, *RORB*, and *NTNG1* showed the most prominent predicted increases in expression (Figure 7C). Following simulated *NRG1* knockout, *AC011288.2*, *PTPRD*, and *RORB* showed prominent predicted increases (Figure 7D), whereas simulated *C3AR1* knockout was associated with predicted expression changes involving *RGS12*, *CXCL14*, and *ADARB2* (Figure 7E). *RORB* and *NTNG1* were among the shared genes showing predicted transcriptional responses to virtual perturbation of *CXCL2* and *NRG1*. In addition, *PTPRD*, *CPNE4*, and *TRHDE* were identified among genes showing computationally predicted responses across the perturbation analyses. These in silico findings are hypothesis-generating and suggest potential transcriptional associations between the hub genes and genes involved in neuronal development, synaptic plasticity, neurotransmission, calcium signaling, and cell adhesion.

### 2.7. Animal Experiment Validation of Hub Gene Expression Changes

**Figure 8:**
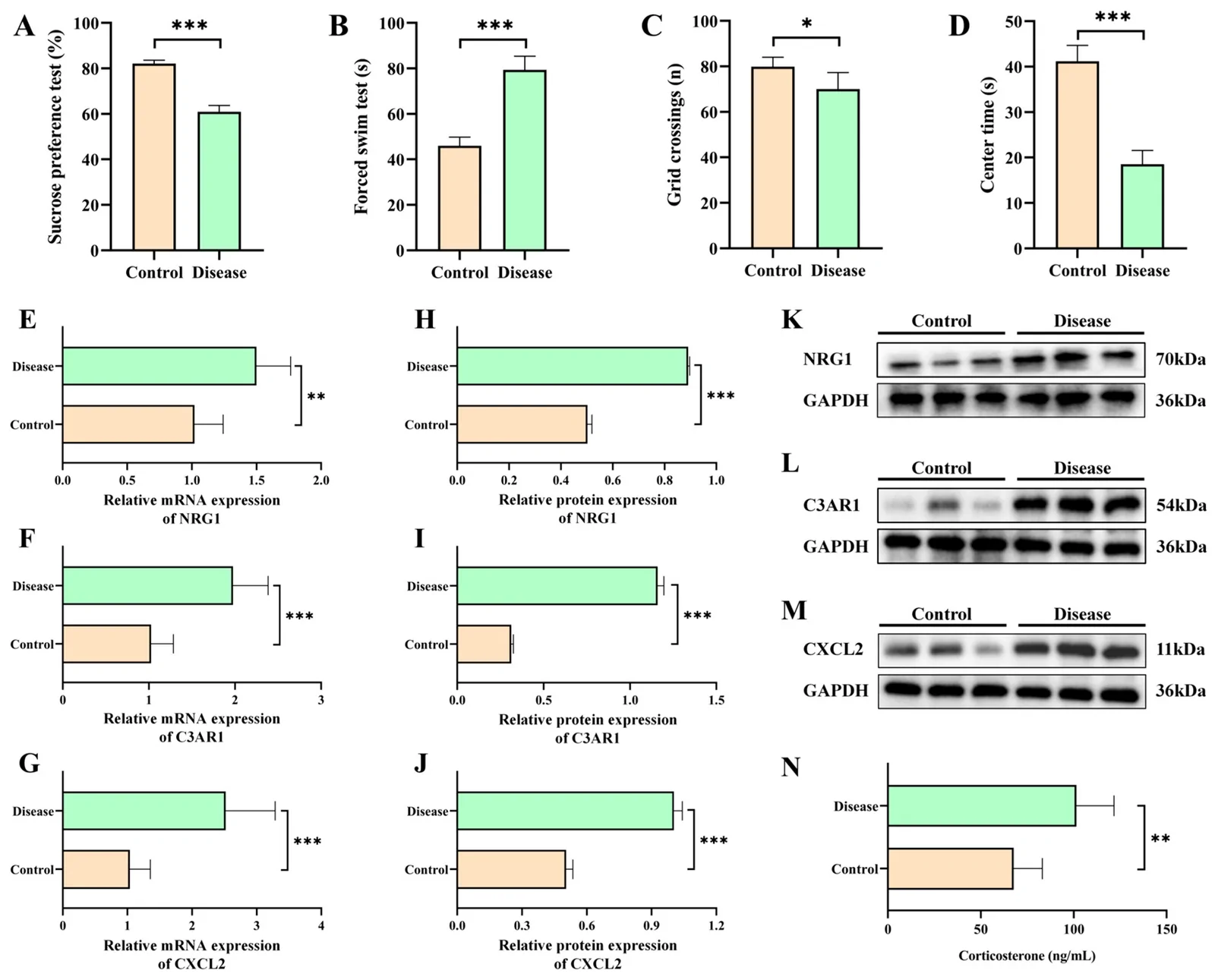
**Animal experiment validation of hub gene expression changes in the PFC of CUMS model rats.** (**A**) Sucrose preference rate of rats after modeling; (**B**) Forced swim test in the forced swimming test; (**C**) Total number of grid crossings in the open field test; (**D**) Central zone residence time in the open field test; (**E**) NRG1 mRNA expression level; (**F**) C3AR1 mRNA expression level; (**G**) CXCL2 mRNA expression level; (**H**) NRG1 protein expression level; (**I**) C3AR1 protein expression level; (**J**) CXCL2 protein expression level; (**K**) NRG1 Western blot bands; (**L**) C3AR1 Western blot bands; (**M**) CXCL2 Western blot bands; (**N**) Serum corticosterone concentration. For behavioral assessments, qRT-PCR analyses, and corticosterone measurements, *n* = 6 per group, where n represents independent biological animals. Each biological sample was subjected to qRT-PCR in triplicate technical replicates, and the averaged values were used. Western blot analysis was performed using *n* = 3 independent biological animals per group randomly selected from the corresponding six-animal cohorts. Normality was assessed using the Shapiro–Wilk test, and no significant departures from normality were detected for the quantitative variables shown in panels (**A**–**J**,**N**). Comparisons between the Control and CUMS groups were therefore performed using two-sided independent-samples *t*-tests (parametric). Compared with the Control group, * indicates *p* < 0.05, ** indicates *p* < 0.01, and *** indicates *p* < 0.001.

Behavioral test results showed that, compared with the Control group, the sucrose preference rate was significantly decreased in the CUMS model group (*p* < 0.001) (Figure 8A). In the forced swimming test, the immobility time was significantly prolonged in the CUMS model group (*p* < 0.001) (Figure 8B). Open field test results demonstrated that the total number of grid crossings was significantly reduced in the CUMS model group (*p* < 0.05) (Figure 8C), and the central zone residence time was significantly shortened (*p* < 0.001) (Figure 8D, Table S3). Taken together, these behavioral results indicated that CUMS treatment induced anhedonia, increased passive coping behavior, decreased spontaneous exploratory activity, and enhanced anxiety-like behavior in rats. In addition, serum corticosterone levels were significantly higher in the CUMS group than in the Control group (*p* < 0.01) (Figure 8N), indicating dysregulation of the hypothalamic-pituitary-adrenal (HPA) axis. The combination of behavioral abnormalities and elevated corticosterone levels provided both behavioral and neuroendocrine evidence supporting successful establishment of the CUMS-induced depression-like model. qRT-PCR results showed (Figure 8E–G) that compared with the Control group, the relative mRNA expression levels of NRG1, C3AR1, and CXCL2 in the PFC of the CUMS model group were significantly elevated (*p* < 0.01). Western blot analysis revealed (Figure 8H–M) that compared with the Control group, the relative protein expression levels of NRG1, C3AR1, and CXCL2 in the PFC of the CUMS group were higher than those in the Control group (*p* < 0.001). To further assess the concordance between the transcript-level and protein-level findings within the same animals, we additionally reanalyzed the qRT-PCR data using only the three animals per group that had been randomly selected for Western blot analysis. In this matched subset, the relative mRNA expression levels of NRG1, C3AR1, and CXCL2 remained significantly higher in the CUMS group than in the Control group (*p* < 0.01) (Figure S1A–C). Thus, all three genes retained the same direction of change observed in the full qRT-PCR cohort and in the Western blot analysis, supporting transcript-protein concordance within the same biological subset.

## 3. Discussion

Major depressive disorder (MDD) is a common and highly disabling psychiatric illness whose pathogenesis involves neuroinflammation, impaired neuroplasticity, and neural circuit dysfunction [7,20]. In recent years, the pivotal role of immune-neural interactions in MDD pathogenesis has received increasing attention. The directed migration of peripheral immune cells toward the central nervous system via a compromised blood–brain barrier [26,27] is considered a core link driving neuroinflammatory cascades in MDD. Cell chemotaxis, as the fundamental biological process by which immune cells undergo directed migration under the guidance of external chemical signals, plays a central regulatory role in inflammatory responses and immune surveillance, and has become a key mechanism connecting peripheral immune activation with central neuroinflammation [28,29,30]. However, systematic studies on the expression characteristics, cell type specificity, and clinical significance of GOBP_TAXIS-related genes in MDD remain lacking. This study integrated microarray-based transcriptomic data with single-cell RNA sequencing data to identify three chemotaxis-related hub genes in MDD (*CXCL2*, *NRG1*, and *C3AR1*) and further explored their diagnostic value, immune microenvironment associations, and cell-type-specific expression characteristics.

*CXCL2* and *NRG1* are core molecules in chemotaxis regulation, and their synergistic dysregulation warrants particular attention. *CXCL2* is a member of the *CXC* chemokine family that mediates the chemotactic recruitment of neutrophils and monocytes through binding to its receptor *CXCR2*, playing a key role in acute inflammatory responses [31,32]. Previous studies have demonstrated that *CXCL2* is upregulated in various neuroinflammatory diseases and can promote immune cell infiltration into the central nervous system, exacerbating neurotoxic damage [33,34]. In this study, *CXCL2* was significantly upregulated in MDD and showed significant positive correlations with monocyte lineage cells, neutrophils, and activated mast cells, as well as negative correlations with T cell subpopulations. These associations are consistent with a potential link between *CXCL2*-related signaling and immune alterations in MDD. *NRG1* is an important neurotrophic factor belonging to the epidermal growth factor family that exerts multiple effects on neural development, synaptic plasticity, myelination, and glial cell function regulation through binding to ErbB receptor tyrosine kinases [35]. In recent years, astrocyte dysfunction has been considered a core element in MDD pathogenesis, with chronic stress inducing reactive astrocyte alterations that lead to glutamate homeostasis imbalance and exacerbated neuroinflammation [36,37]. This study found that *NRG1* was widely and highly expressed in astrocyte subclusters and showed a positive correlation with monocytes and a negative correlation with T cell subpopulations, suggesting a potential association of *NRG1* with astrocyte-related and immune features in MDD. Notably, in silico knockout simulations identified *RORB* and *NTNG1* among the shared genes showing predicted transcriptional responses to virtual perturbation of *CXCL2* and *NRG1*. These computational findings suggest a potential association between *CXCL2/NRG1* and transcriptional programs related to neuronal development and synaptic plasticity.

*C3AR1* is a key receptor of the complement system, and its dual role in immune regulation merits in-depth discussion. *C3AR1* encodes the receptor for complement C3a, belonging to the G protein-coupled receptor family, and is widely expressed in microglia and peripheral monocytes/macrophages, participating in the fine regulation of neuroinflammation [20]. Previous studies have indicated that the *C3AR1* signaling pathway exerts opposing functions in the central and peripheral immune systems: microglial *C3AR1* activation promotes stress susceptibility, whereas peripheral macrophage *C3AR1* exerts a protective effect [20]. In this study, *C3AR1* showed significant positive correlations with monocytes and neutrophils, and was extensively associated with various inflammatory factors such as *TNF* and *IL-15*. These associations suggest that *C3AR1* expression is linked to immune-cell abundance patterns and inflammatory signatures in MDD, although its functional role in neuroinflammation requires direct experimental validation. However, *C3AR1* expression was relatively dispersed in single-cell analysis, with low-level expression detectable in macrophages and partial astrocytes, and its cell type-specific functions require further experimental validation.

In immune subtype analysis, consensus clustering revealed significant heterogeneity in the MDD immune microenvironment. Consensus clustering analysis based on the three hub genes stratified MDD samples into two subtypes, Cluster 1 and Cluster 2. There were no significant differences in gender or age distribution between the two subtypes, suggesting that this classification primarily reflected molecular characteristic differences rather than demographic bias. Cluster 1 was characterized by high *C3AR1* expression and low *CXCL2* and *NRG1* expression, accompanied by elevated monocyte lineage and neutrophil abundance. Cluster 2 showed the opposite pattern, with significantly elevated T cell abundance. WGCNA analysis showed that the MEgrey60 module was significantly positively correlated with Cluster 1, and genes in this module were mainly enriched in antigen processing and presentation, regulation of adaptive immune response, and lysosome-related functions. These findings indicate that molecular subtypes characterized by different hub-gene expression patterns are associated with distinct computationally estimated immune-cell abundance profiles. Cluster 1 was associated with higher monocytic-lineage and neutrophil abundance, whereas Cluster 2 showed higher T-cell abundance. Previous studies have similarly found that abnormalities in the chemokine-receptor signaling axis can lead to significant differences in immune cell infiltration levels, and MDD subgroups with high chemokine expression often accompany pro-inflammatory immune cell-dominant phenotypes [28,38]. Notably, this study further validated these findings at the single-cell level. *NRG1* was mainly highly expressed in astrocytes, *CXCL2* was enriched in endothelial cells and astrocyte subclusters, and *C3AR1* showed low-level expression in macrophages. This cell type-specific distribution pattern indicates that different chemotaxis-related genes can participate in MDD immune regulation by influencing specific microenvironmental cellular components.

Single-cell analysis revealed the fine distribution atlas of *CXCL2*, *NRG1*, and *C3AR1* hub genes in the MDD brain tissue microenvironment. Four major cell types were identified, including astrocytes, endothelial cells, macrophages, and neuroendocrine cells, with the astrocyte subpopulation being the absolutely dominant group. Disease-control comparison showed that endothelial cells and neuroendocrine cells were significantly enriched in the control group with reduced proportions in the MDD group, while astrocytes and macrophages showed similar proportions between the two groups, indicating relative reduction of endothelial cells and neuroendocrine cells in MDD patients and relatively stable astrocyte proportions. Hub genes were mainly highly expressed in astrocytes and endothelial cells, with relatively low expression in macrophages and neuroendocrine cells. This distribution pattern suggests that chemotaxis-related genes primarily participate in MDD pathogenesis by influencing neural parenchymal cells (astrocytes) and key blood–brain barrier components (endothelial cells).

Additionally, the in silico gene knockout simulations predicted distinct transcriptional responses following virtual perturbation of *CXCL2*, *NRG1*, and *C3AR1*. *RORB* and *NTNG1* were among the shared genes showing predicted responses to *CXCL2* and *NRG1* perturbation, while *PTPRD*, *CPNE4*, and *TRHDE* were also implicated in the computational perturbation analyses. Because these findings were derived entirely from transcriptome-based simulation, they should be interpreted as candidate associations for subsequent experimental validation rather than evidence of direct downstream regulation.

An important translational consideration is that the upstream bioinformatic analyses were based on human transcriptomic datasets, whereas experimental validation was performed in a CUMS rat model. The CUMS paradigm was selected because chronic unpredictable stress induces reproducible depression-relevant behavioral, neuroendocrine, synaptic, and neuroimmune alterations and is widely used to investigate stress-related mechanisms of depression. The purpose of the animal experiment in the present study was therefore not to reproduce the complete human MDD transcriptomic landscape, but to examine whether the human-derived candidate genes showed concordant stress-associated expression in an independent in vivo mammalian system. Cross-species transcriptomic studies have shown that direct overlap of individual differentially expressed genes between human MDD and rodent chronic-stress models can be limited, whereas greater conservation may emerge at the pathway, regulatory-network, and functional-module levels [39]. Accordingly, the present rat data should be interpreted as supportive cross-species biological validation rather than evidence of complete molecular equivalence between human MDD and the CUMS model.

Gene-specific evidence also provides biological support for this cross-species strategy. For *NRG1*, previous work in CUMS-exposed rats demonstrated dysregulation of *NRG1* signaling and increased *NRG1* expression in the prefrontal cortex, consistent with the direction observed in our rat validation [40]. For the complement pathway, human and rodent studies have implicated C3/C3aR signaling in depression-related neuroimmune responses: increased C3 expression has been reported in the PFC of depressed individuals, while chronic stress in mice induces C3aR-dependent monocyte recruitment and depressive-like behavior, and C3aR deficiency attenuates these effects [41]. CUMS studies have also reported activation of C3/C3aR signaling in the PFC during the development of depression-like behavior [42]. With respect to *CXCL2*, rodent stress models support the involvement of *CXCL2*-related chemokine signaling in stress-associated neuroinflammatory responses, including increased *CXCL2*-related signals in CUMS rats; however, the direction and magnitude of *CXCL2* alterations appear to vary across species, tissues, and experimental contexts [43,44]. Thus, the concordant upregulation observed in our CUMS rat PFC provides supportive evidence for the biological relevance of the three candidates, but additional validation in independent human brain and peripheral cohorts remains necessary.

HPA-axis dysregulation is a well-established neuroendocrine feature associated with MDD. In rodents, corticosterone represents the principal circulating glucocorticoid and is commonly used as an indicator of HPA-axis activity. In the present study, CUMS-exposed rats exhibited significantly elevated serum corticosterone levels compared with Control rats. Together with the reduced sucrose preference, increased immobility in the forced swim test, and impaired exploratory behavior in the open field test, this neuroendocrine alteration provides additional support for the validity of the CUMS model used for subsequent molecular validation.

Despite the integration of multi-omics data to reveal the expression characteristics and clinical significance of chemotaxis-related hub genes in MDD, this study has certain limitations. First, data were derived from public databases, and the retrospective design carries limitations including batch effects, sample heterogeneity, and incomplete clinical information, which may introduce potential bias and affect result reproducibility. Second, single-cell annotation relies on known marker genes, and the limited sample size of the dataset may affect the completeness of cell subpopulation identification. Third, this study focused primarily on the transcriptional level and did not involve epigenetic regulatory mechanisms such as DNA methylation and non-coding RNAs. Fourth, although the diagnostic model performed robustly in Bootstrap internal validation, it lacked validation in independent external datasets, which may affect the model’s generalizability. Fourth, the model was trained on a small cohort (21 controls and 29 MDD cases) with only internal bootstrap validation. Because feature selection was not nested within each resampling iteration, the uncertainty introduced by this step was not fully captured. Therefore, the AUC of 0.889 should be regarded as an exploratory estimate rather than definitive evidence of diagnostic performance. Fifth, the single-cell knockout analysis was an in silico computational perturbation performed using scTenifoldKnk rather than an experimental gene knockdown assay. Therefore, the predicted transcriptional responses should be interpreted as hypothesis-generating associations and require validation using genetic or pharmacological perturbation experiments. Sixth, as the dataset was derived from different tissues (peripheral blood, human DLPFC, and rat PFC), peripheral expression may not directly reflect brain expression, and further evaluation of cross-tissue consistency is required. Several limitations of the animal experiment should also be acknowledged. First, molecular validation was restricted to the prefrontal cortex (PFC), without examining other depression-related brain regions such as the hippocampus or amygdala. Second, we confirmed the upregulation of hub genes at the transcriptional and protein levels but did not establish causality through genetic or pharmacological interventions. In addition, the candidate genes were initially identified from human transcriptomic datasets, whereas molecular validation was performed in the PFC of CUMS rats. Species differences, tissue-specific expression patterns, and differences in cellular composition may limit direct extrapolation from the rat model to human MDD. Therefore, the animal experiment should be considered supportive cross-species validation rather than direct replication of the human transcriptomic findings. Future studies using independent human cohorts, postmortem brain tissue, and cross-species transcriptomic comparisons are needed to further establish translational concordance. At the same time, future research should validate the predictive performance of the diagnostic model in multicenter independent cohorts and assess its clinical translational value, elucidate the molecular mechanisms by which hub genes regulate immune cell migration through chemotaxis experiments and gene editing techniques, and combine spatial transcriptomics technology to resolve the spatial expression patterns of chemotaxis-related genes in the central nervous system and their spatial associations with immune infiltration.

## 4. Methods

### 4.1. Data Collection

Two microarray datasets (GSE76826 and GSE38206) were retrieved from the GEO database using MDD as the search term. Sample data from healthy controls and MDD cases were extracted. Both datasets were derived from peripheral blood samples. Specifically, GSE76826 profiled gene expression in peripheral blood cells from individuals with major depressive disorder and healthy controls, whereas GSE38206 profiled peripheral blood mononuclear cells (PBMCs) from patients with major depressive episodes and matched controls. Samples corresponding to the MDD and control groups were extracted for the present analysis. The chemotaxis-related gene set (GOBP_TAXIS, https://www.gsea-msigdb.org/gsea/msigdb/human/geneset/GOBP_TAXIS, 5 August 2026) was obtained from the GSEA-MSigDB database. Dataset identity was used as the batch variable, and batch effects were removed using the removeBatchEffect function in the “limma” package in R version 4.3.3 [45]. The batch-corrected integrated expression matrix comprised 21 control samples and 29 MDD samples. Principal component analysis (PCA) was performed before and after batch-effect correction to visualize sample distributions and assess the effectiveness of batch correction. Differential expression analysis between the MDD and control groups was performed exclusively using the “limma” package. A design matrix was constructed according to disease status, and the MDD-versus-control contrast was fitted using lmFit, followed by contrasts.fit and empirical Bayes moderation using eBayes. Differential-expression statistics were obtained using topTable, with *p* values adjusted for multiple testing using the false discovery rate (FDR) method. Genes with an absolute log2 fold change (|log2FC|) > 0.5 and an FDR-adjusted P value < 0.05 were considered differentially expressed.

### 4.2. Construction of the MDD Diagnostic Model

The DEGs identified from the integrated microarray dataset were intersected with the GOBP_TAXIS gene set obtained from the Molecular Signatures Database (MSigDB) to identify GOBP_TAXIS-related candidate genes. For gene set enrichment analysis, the ssGSEA algorithm from the “gsva” package was applied, and enrichment results were visualized using the “ggpubr” package. To evaluate the significance of GOBP_TAXIS-related genes (GTAGs) in MDD, random forest and support vector machine recursive feature elimination (SVM-RFE) were employed for feature selection. SVM-RFE incorporated 10-fold cross-validation to rank candidate features and determine the optimal number of features, whereas random forest feature importance was estimated using the complete candidate-gene dataset. The overlapping genes identified by the two algorithms were then selected as the fixed candidate hub genes for subsequent multivariable logistic regression analysis. The diagnostic performance of the resulting three-gene model was evaluated using receiver operating characteristic (ROC) curve analysis. Bootstrap resampling was subsequently performed as an internal assessment of the discrimination of this fixed three-gene model. Importantly, the upstream feature-selection procedures, including SVM-RFE and random forest screening, were completed before bootstrap evaluation and were not repeated within each bootstrap resampling iteration. Therefore, the bootstrap procedure assessed the fitted model conditional on the preselected feature set rather than providing nested validation of the entire feature-selection pipeline. Given the relatively small development cohort and the absence of an independent external validation cohort, the resulting AUC should be interpreted as an exploratory internally assessed estimate that may be subject to optimism. On this basis, a nomogram and calibration curve were constructed, and decision curve analysis was performed to explore the potential net benefit of the model within the development cohort. For the expression comparisons of the three hub genes between the Control and MDD groups, two-sided Welch’s t-tests were used. Multivariable logistic regression coefficients were evaluated using Wald tests, and odds ratios (ORs) with 95% confidence intervals (CIs) were reported.

### 4.3. Association Between Hub Gene Expression and Computationally Estimated Immune-Cell Abundance

Immune-cell deconvolution analyses were performed to investigate associations between hub-gene expression and computationally estimated immune-cell abundance. The CIBERSORT algorithm implemented in the “IOBR” package was used to estimate the relative proportions of 22 immune-cell types, and correlations between these estimates and hub-gene expression were calculated. The MCP-counter algorithm was additionally used to estimate the abundance scores of selected immune-cell populations and assess their correlations with hub-gene expression. Associations between hub-gene expression and 28 inflammation-related factors were also evaluated. These computational analyses were used to assess statistical associations and were not interpreted as direct measurements of immune-cell migration or tissue infiltration.

### 4.4. Consensus Clustering and Multi-Clinical Index Comparison

Consensus clustering analysis was performed using the “ConsensusClusterPlus” package. Genes were ranked by median absolute deviation, and the top 3000 most variable genes were selected for unsupervised clustering to determine the optimal cluster number [46]. During clustering, Ward’s method was used as the linkage approach, Euclidean distance as the similarity metric, and more than 1000 resampling iterations were conducted. To further explore gene co-expression patterns, the weighted gene co-expression network analysis (WGCNA) R package was employed to identify synergistically changing mRNA modules in MDD samples [47]. The expression matrix was first standardized and then transformed into an adjacency matrix using a power adjacency function to measure the connection strength between genes. The soft thresholding power was selected based on scale-free topology criteria to ensure a model fitting degree exceeding 0.85, and the “pickSoftThreshold” function ultimately determined the soft thresholding power as 5. Module detection was performed using hierarchical clustering (the “tree” method) combined with the “deepSplit” parameter. Only modules containing no fewer than 10 genes were retained, and the eigengene value of each module was calculated to summarize its expression profile. For functional annotation of modules, the Gorilla tool was used to perform Gene Ontology (GO) biological process enrichment analysis on the identified WGCNA modules [48]. Simultaneously, functional characterization of the clustering results was conducted based on 50 hallmark gene sets from the Molecular Signatures Database (MSigDB) using gene set enrichment analysis (GSEA), with normalized enrichment score (NES) > 1 and *p* < 0.01 as the statistical significance threshold [49].

### 4.5. Functional Enrichment Analysis of Key Module Genes

GO and KEGG over-representation analyses were performed using the clusterProfiler package. Gene symbols were converted to Entrez Gene identifiers using the org.Hs.eg.db annotation package. GO enrichment was separately performed for biological process (BP), cellular component (CC), and molecular function (MF) categories using enrichGO, whereas KEGG pathway enrichment was performed using enrichKEGG with Homo sapiens specified as the target organism. Enrichment significance was evaluated using the hypergeometric test, with P values adjusted for multiple comparisons using the Benjamini–Hochberg procedure.

### 4.6. Single-Cell Analysis

The MDD single-cell dataset was downloaded from GSE144136 and subjected to batch quality control. In contrast to the peripheral blood-derived microarray discovery datasets, GSE144136 was generated from nuclei isolated from postmortem dorsolateral prefrontal cortex tissue of 17 control subjects and 17 individuals with MDD. The “Seurat” package was used to analyze single-cell RNA sequencing data. First, cell filtering was performed based on mitochondrial (MT) and hemoglobin (HB) gene expression to ensure data quality. The specific quality control criteria were as follows: the number of genes detected per cell (nFeature_RNA) ranged between 300 and 7000; the UMI count per cell (nCount_RNA) exceeded 1000, excluding the top 3% of cells with the highest counts; the proportion of mitochondrial gene expression per cell (mt_percent) was kept below 10%; and the proportion of hemoglobin gene expression per cell (HB_percent) was kept below 3%. The gene expression matrix was initially normalized using the NormalizeData function to reduce differences in expression levels between genes. Subsequently, the normalized data were standardized using the ScaleData function to ensure a uniform baseline for comparison in subsequent analyses. The RunPCA function was then used to calculate principal components (PCs) of the data. Finally, the Harmony method was applied to eliminate batch effects. The FindAllMarkers function was used to analyze normalized gene expression data with Seurat default parameters. The standard FindNeighbors function was then used for clustering, considering the first 13 principal components. Subsequently, the FindClusters function was used for clustering with the resolution parameter set at 0.8. Finally, the UMAP method was applied for dimensionality reduction.

For in silico gene perturbation analysis, single-cell gene knockout simulations were performed using the scTenifoldKnk R package. For each hub gene, the raw count matrix was restricted to the target gene together with the top 2000 highly variable genes identified using the vst method. Virtual knockout was then performed separately for *CXCL2*, *NRG1*, and *C3AR1* using scTenifoldKnk with the following parameters: qc_mtThreshold = 0.1, qc_minLSize = 1000, nc_nNet = 3, and nc_nCells = 300. Differential regulation statistics generated by scTenifoldKnk were extracted from the simulated knockout results, and genes with adjusted *p* values < 0.05 were considered to show significant predicted transcriptional responses. These analyses were entirely computational and did not involve experimental gene knockdown or gene editing.

### 4.7. Animal Experiment Validation

#### 4.7.1. Experimental Animals, Model Establishment, and Sample Collection

##### Experimental Animals

SPF male Sprague–Dawley rats (*n* = 12), 8 weeks old, weighing 180 to 220 g, were purchased from Hangzhou Medical College [license number SCXK (Su) 2024-0001]. After arrival, the animals were allowed to acclimatize for 7 days with free access to standard chow and tap water. The housing environment was maintained at a temperature of 21 ± 2 °C and a relative humidity of 40% to 60%, with a 12-h light/dark cycle (lights on from 08:00 to 20:00). Animals were housed in standard polypropylene cages (three per cage) with wood chip bedding, which was changed twice weekly. Exclusion criteria were established a priori: animals presenting with overt illness upon arrival, failure to gain weight during acclimatization, severe injury from inter-animal aggression, or extreme behavioral anomalies during testing were excluded. Rats were randomly assigned to the Control group (*n* = 6) or the CUMS group (*n* = 6) using a random number table. The individual rat was considered the experimental unit for all animal experiments and statistical analyses. All six animals in each group were included in the behavioral assessments and qRT-PCR analysis. For Western blot analysis, three animals were randomly selected from the six animals in each group as biological replicates.

##### CUMS Procedure and Behavioral Assessments

The CUMS group was subjected to chronic unpredictable mild stress for 21 consecutive days. Each day, one or two of the following stressors were randomly applied, including food deprivation for 24 h, water deprivation for 24 h, reversal of the light/dark cycle, wet bedding, empty cage, cage tilt, restraint for 2 h, cold water swimming, and tail pinch. The Control group was maintained under standard housing conditions without any stress exposure. Following model establishment, depressive-like behaviors were evaluated sequentially by the sucrose preference test (SPT), open field test (OFT), and forced swim test (FST), with a 24-h interval between two consecutive tests. For the SPT, after 24 h of food and water deprivation, each rat was simultaneously presented with one bottle of preweighed 1% sucrose solution and one bottle of pure water for a 1 h test. The positions of the two bottles were exchanged at 30 min during the test period. The sucrose preference rate (%) was calculated as [sucrose intake / (sucrose intake + pure water intake)] × 100%. For the OFT, each rat was individually placed in the center of an open field arena (100 × 100 × 40 cm) and allowed to explore freely for 5 min under 200 to 300 lux illumination. The central zone was defined as the central 25% of the total arena area. The number of grid crossings and the time spent in the central zone were recorded. After each test, the arena was thoroughly cleaned with 75% ethanol and dried to eliminate odor residues. For the FST, on day 1, rats were placed individually in a transparent cylindrical tank (30 cm diameter, 50 cm height) filled with water (23 ± 1 °C) to a depth of 30 cm for a single 5-min test session. Immobility was defined as floating with only minimal movements necessary to keep the head above water, excluding active swimming or climbing. The water was changed after each rat. All behavioral assessments were performed by the same investigator. The cages were randomly allocated on the shelving racks, and all animals were maintained at the same housing density throughout the experimental period.

##### Tissue Collection and Ethical Compliance

Twenty-four hours after the behavioral tests, the animals were anesthetized and euthanized. Specifically, 3% pentobarbital sodium (30 mg/kg, 0.1 mL/100 g; Sigma-Aldrich, St. Louis, MO, USA) was administered by intraperitoneal injection. Deep anesthesia was confirmed by loss of the righting reflex, slow and shallow breathing, and absence of response to toe pinch. Blood was collected through the abdominal aorta, followed by exsanguination. This procedure conforms to the American Veterinary Medical Association (AVMA) Guidelines for the Euthanasia of Animals regarding rodents. Pentobarbital sodium-induced deep anesthesia ensures unconsciousness at death, and exsanguination serves as an adjunct to ensure definitive death and prevent potential anesthetic recovery. Animals were monitored daily for general appearance, food and water intake, and body weight. Immediate euthanasia was performed if any of the following signs were observed: (1) body weight loss exceeding 20% or appearance of cachexia; (2) complete anorexia for 24 h or food intake below 50% of normal for 3 consecutive days; or (3) inability to eat, drink, or stand for 24 h without sedation. Immediately following euthanasia, the prefrontal cortex (PFC) was rapidly dissected on ice, snap-frozen in liquid nitrogen, and stored at −80 °C until further analysis. The collected PFC tissue was subsequently used for both qRT-PCR and Western blot analyses. The study protocol was reviewed and approved by the Experimental Animal Ethics Committee of Nanjing University of Chinese Medicine (approval number 202411A068). All animal experiments were conducted in accordance with the ARRIVE guidelines and the regulations of the Institutional Animal Care and Use Committee of Nanjing University of Chinese Medicine. All animals were sourced from a licensed commercial breeder, and no privately owned or personally sourced animals were used in this study. Accordingly, no additional informed consent was necessary. Investigators administering the CUMS procedures were aware of group allocation because of the nature of the intervention. Behavioral testing, molecular assays, and data analysis were performed by independent researchers blinded to group allocation using coded samples.

#### 4.7.2. Quantitative Real-Time PCR

Total RNA was extracted from frozen PFC tissue using the TRIzol method. RNA concentration and purity were assessed with a micro-spectrophotometer (NanoDrop One; Thermo Fisher Scientific, Waltham, MA, USA), and samples with an A260/A280 ratio of 1.8–2.0 were used for subsequent experiments. Equal amounts of total RNA were reverse-transcribed into cDNA using a reverse transcription kit (Vazyme, Nanjing, China) according to the manufacturer’s instructions. The mRNA expression of NRG1, C3AR1, and CXCL2 was detected using a SYBR Green real-time quantitative PCR system, with GAPDH as the internal reference. The 20 μL reaction mixture contained 10.0 μL of 2× SYBR Green Mix (Yeasen, Shanghai, China), 0.4 μL each of forward and reverse primers, 2.0 μL of cDNA, and 7.2 μL of nuclease-free water. The amplification conditions were pre-denaturation at 95 °C for 30 s, followed by 40 cycles of 95 °C for 5 s and 60 °C for 30 s. A melting curve analysis was performed after the reaction. qRT-PCR analysis was performed using PFC samples from all six animals in each group (*n* = 6 independent biological replicates per group). Each biological sample was measured in triplicate technical replicates. The triplicate measurements were averaged to obtain a single value for each animal, and these averaged biological values were used for statistical analysis. Primer sequences are listed in Table S1.

#### 4.7.3. Western Blot

Western blot analysis was performed using PFC tissue from three biological replicates per group (*n* = 3/group), randomly selected from the six animals in each experimental group. Each lane represented an independent biological sample from one rat. Frozen PFC tissue was homogenized in RIPA lysis buffer (Beyotime, Shanghai, China) containing protease inhibitors (Beyotime, Shanghai, China) and centrifuged at 12,000× *g* for 15 min at 4 °C. The supernatant was collected, and protein concentration was determined using the BCA protein assay kit (Beyotime, Shanghai, China). Equal amounts of total protein (25 μg) from each sample were separated by 10–15% SDS-PAGE and transferred onto PVDF membranes (Merck Millipore, Burlington, MA, USA). The membranes were blocked with 5% skim milk at room temperature for 1 h, then incubated overnight at 4 °C with primary antibodies against NRG1 (Novus Biologicals, Centennial, CO, USA), C3AR1 (Elabscience, Wuhan, China), CXCL2 (Thermo Fisher Scientific, Waltham, MA, USA), and GAPDH (Abcam, Cambridge, UK). After washing with TBST, the membranes were incubated with HRP-conjugated secondary antibodies (Proteintech, Wuhan, China) at room temperature for 1 h. Protein bands were visualized using ECL chemiluminescence reagent (Yeasen, Shanghai, China) and captured with an imaging system (Tanon, Shanghai, China). GAPDH served as the loading control. Band intensities were quantified using ImageJ 1.54p software, and relative protein expression was calculated as the ratio of target protein band intensity to GAPDH band intensity. Antibody information is provided in Table S2.

#### 4.7.4. Serum Corticosterone Measurement

Serum corticosterone (CORT), the principal circulating glucocorticoid in rats, was measured as an indicator of HPA-axis activity. Blood samples collected from the abdominal aorta were allowed to clot and centrifuged at 3000 rpm for 10 min at 4 °C. The separated serum was stored at −80 °C until analysis. Serum corticosterone (CORT) concentrations were measured using a commercially available rat corticosterone ELISA kit (Aoqing Biotechnology, Shanghai, China, catalog No. ANG-E51003R) according to the manufacturer’s instructions. Samples from all animals in the Control and CUMS groups were analyzed (*n* = 6 independent biological animals per group). Each sample was measured in triplicate technical replicates, and the averaged value for each animal was used for statistical analysis.

#### 4.7.5. Statistical Analysis

Animal experimental data were analyzed using SPSS 27.0 (IBM Corp., Armonk, NY, USA) and GraphPad Prism 8.4.3 (GraphPad Software, San Diego, CA, USA). Continuous variables with normal distribution confirmed by the Shapiro–Wilk test were expressed as mean ± standard deviation, and comparisons between the two groups were performed using an independent samples t-test. Variables that did not follow a normal distribution were analyzed using the Mann–Whitney U test. All tests were two-tailed, and a P value less than 0.05 was considered statistically significant.

## 5. Conclusions

This study integrated microarray-based transcriptomic data with single-cell RNA sequencing data to reveal the expression characteristics of chemotaxis (GOBP_TAXIS)-related genes in MDD and their associations with immune microenvironment remodeling. The study confirmed that *CXCL2*, *NRG1*, and *C3AR1* were significantly upregulated in MDD. The three-gene model showed preliminary discriminatory ability in the development cohort (AUC = 0.889). Computational immune-cell deconvolution analyses showed that *CXCL2*, *NRG1*, and *C3AR1* expression was associated with estimated monocytic-lineage, neutrophil, and T-cell abundance, as well as with several inflammation-related factors. Single-cell analysis further clarified the predominant expression of *NRG1* in astrocytes, enrichment of *CXCL2* in endothelial cells, and distribution characteristics of *C3AR1* in macrophages, suggesting that chemotaxis-related genes may participate in the neuroinflammatory pathological process of MDD through intercellular signaling interactions among different cell types. These findings provide novel insights into the immune-chemotaxis mechanisms of MDD and provide candidates for further investigation into immune-chemotaxis mechanisms and biomarker development in MDD.

## Data Availability

Two microarray datasets, designated GSE76826 and GSE38206, were obtained from the GEO database. The chemotaxis-related gene set (GOBP_TAXIS, https://www.gsea-msigdb.org/gsea/msigdb/human/geneset/GOBP_TAXIS, accessed on 5 August 2026) was downloaded from the GSEA-MSigDB database. All the data generated or analyzed in this study have been incorporated into this article and Supplementary Materials. Further information is available from the corresponding author upon reasonable request.

## Supplementary Materials

The following supporting information can be downloaded at: https://www.mdpi.com/article/10.3390/ijms27198780/s1.
