# Supplementary material for: Cell Tropism-Related Gene Signatures in MDD Identified by Transcriptomic Analysis and Rat Model Validation
Source: Int J Mol Sci. 2026 Sep 30;27(19):8780. doi: 10.3390/ijms27198780 (PMC13664878; doi:10.3390/ijms27198780)
Supplement: Supplementary file 1 [file ijms-27-08780-s001.zip › Supplementary Figure.pdf]

Supplementary Figure

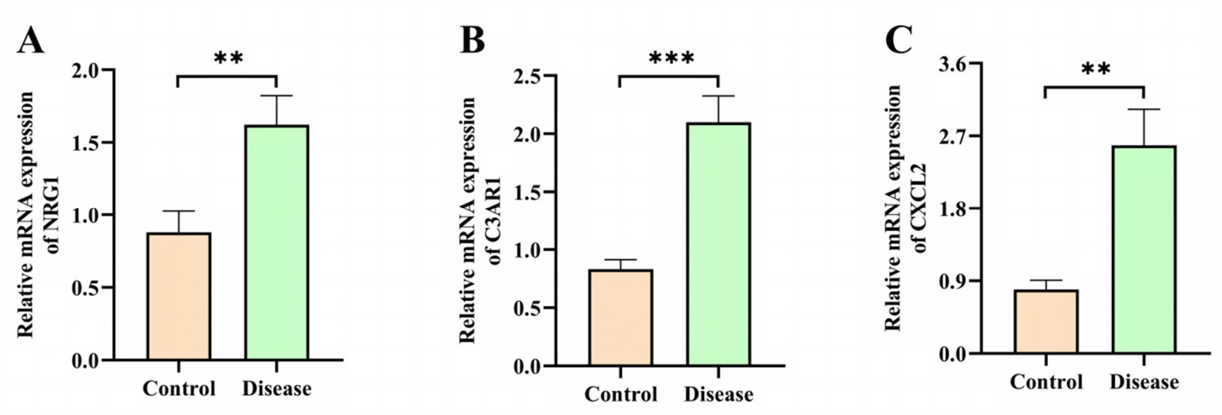

Figure S1 . Matched-subset qRT-PCR analysis of the same animals used for Western blot validation. qRT-PCR data were reanalyzed using the three animals per group that had been randomly selected from the original six-animal cohorts for Western blot analysis. (A) Relative mRNA expression of NRG1; (B) relative mRNA expression of C3AR1; (C) relative mRNA expression of CXCL2. n = 3 independent biological animals per group. Each biological sample was measured in triplicate technical qRT-PCR replicates, and the averaged value was used as one biological observation for statistical analysis. **P < 0.01 and ***P < 0.001 versus the Control group.
