# Supplementary material for: Cell Tropism-Related Gene Signatures in MDD Identified by Transcriptomic Analysis and Rat Model Validation
Source: Int J Mol Sci. 2026 Sep 30;27(19):8780. doi: 10.3390/ijms27198780 (PMC13664878; doi:10.3390/ijms27198780)
Supplement: Supplementary file 1 [file ijms-27-08780-s001.zip › Supplementary Tables.pdf]

Supplementary Tables

Table S1 qRT-PCR primer sequences.

Gene	Forward primer	Reverse primer
NRG1	GCGGAGAAGGAGAAAACTTTC	TTGCTCCAGTGAATCCAGGTTG
C3AR1	CAGGACAAAGCCCCCTCAAA	AGGAGATGGCAAGATCCTCAG
CXCL2	AGGGCGGTCAAAAAGTTTGC	CAGGTACGATCCAGGCTTCC
GAPDH	GCAAGTTCAATGGCACAG	AAGTTCTTCCTGGCCGGATT

Table S2 Western blot antibody information.

Antibody	Source/Company	Catalog number	Dilution ratio
NRG1	Novus Biologicals (Bio-Techne)	NBP2-19587	1:1000
C3AR1	Elabscience	E-AB-93391	1:1000
CXCL2	Thermo Fisher Scientific	PA5-88673	1:500
GAPDH	Abcam	ab22555	1:5 000
secondary antibody	Proteintech	SA00001-2	1:5 000

Table S3 Behavioral characterization of CUMS-induced depressive-like phenotype in rats.

	Control (Mean)	Control (SD)	Disease (Mean)	Disease (SD)	*P*
Sucrose preference test（%）	82.150	1.498	60.917	2.827	0.000
Forced swim test (s)	45.933	3.865	79.364	6.011	0.000
Grid crossings (n)	79.833	4.167	70.000	7.266	0.021
Center time (s)	41.181	3.518	18.528	3.049	0.000
